# A dataset on the multi-species and multi-endpoint bioanalytical assessment of environmental samples derived from Swedish wastewater treatment plants

**DOI:** 10.1016/j.dib.2026.113036

**Published:** 2026-07-02

**Authors:** Sebastian Lungu-Mitea, Geeta Mandava, Oksana Golovko, Kim Frieberg, Sara Alsayfi, Stefan Örn, Lutz Ahrens, Johan Lundqvist

**Affiliations:** a Department of Animal Biosciences, Molecular Toxicology, Swedish University of Agricultural Sciences (SLU), SE-75007, Uppsala, Sweden; b Department of Pharmaceutical Biosciences, Drug Safety and Toxicology, Uppsala University (UU), SE-75124, Uppsala, Sweden; c Department of Biology and Environmental Science, Linnaeus University (LNU), SE-39182, Kalmar, Sweden; d Department of Aquatic Sciences and Assessment, Swedish University of Agricultural Sciences (SLU), SE-75007, Uppsala, Sweden

**Keywords:** Bioanalytical toxicology, Effect-based methods, Fish embryo test, Reporter gene assays, Wastewater assessment

## Abstract

*In vitro* bioanalytical tools show great potential to support analytical chemistry in the high-throughput assessment of environmental samples and are approaching regulatory implementation. This dataset showcases the usage of two *in vitro* systems, zebrafish embryos and cellular reporter gene assays, to assess the toxicological impact of several environmental samples. The samples were taken from three Swedish wastewater treatment plant sites (influent, effluent) and their respective catchments (upstream and downstream recipients). The samples were concentrated via automated solid-phase extraction. Zebrafish embryos were exposed to all environmental samples up to 144 hpf, and sublethal and lethal apical endpoints were recorded in a modified fish embryo acute toxicity test. Influent and effluent samples were further evaluated with five reporter-gene cell lines, covering three different species (zebrafish, human, mouse) and two different modes of action (oxidative stress, xenobiotic metabolism). Cellular viability was assessed from identical technical replicates. All endpoints were evaluated using a no-effect/lowest-effect-concentration (NOEC/LOEC) approach, enabling comparison between whole-organism and cellular *in vitro* systems. In addition, inter-species sensitivity was assessed by comparing differences across reporter-gene assays. This data article supports an original research article, which builds in part on the raw data presented here, making it accessible to a broader audience and sharing raw data in accordance with the FAIR principles. Fish embryo toxicity assessments and the analysis of upstream and downstream recipient samples are exclusive to this data article. As such, the dataset could be of interest to scientists, regulators, and water treatment operators involved in the toxicological assessment of environmental samples.

Specifications Table

**Table:** 

Subject	Biology
Specific subject area	Environmental Toxicology, Bioanalytical assessment of environmental samples
Type of data	Table Chart Figure Raw - within supplementary files Analysed - within the article and supplementary files Illustrative - within the article and supplementary files
Data collection	Samples from water treatment plants (WWTP; influent + effluent) and their recipients (upstream + downstream) were obtained by solid-phase extraction (SPE-DEX, Horizon Technology). All samples were bioanalytically assessed by a modified zebrafish embryo test, scoring lethal and sublethal endpoints via stereo microscopy (Leica EZ4D) and time-lapse camera recording (Canon EOS 500D). Influent and effluent samples were analysed by reporter gene assays of three different species (zebrafish, human, mouse), covering two modes of action: oxidative stress and xenobiotic metabolism. Cellular viability was assessed via the MTS assay. All endpoints were recorded on a plate reader (Spark, Tecan).
Data source location	Institution: Swedish University of Agricultural Sciences (SLU) City, Region: Uppsala, Uppland Country: Sweden
Data accessibility	Repository name: Swedish National Data Service (SND): The SND Data Organisation and Information System (Doris) Data identification number: 2025–199; doi.org/10.5878/zwnq-qb88 Direct URL to data: https://doi.org/10.5878/zwnq-qb88
Related research article	DOI:10.1016/j.ecoenv.2026.120709

## 1 Value of the Data

• The dataset can be used to estimate the WWTP's removal efficiency and potential environmental burden on the downstream biosphere.
• Further, assessment of pollutants of emerging concern via effect-directed analysis, particularly when combined with analytical chemistry.
• The dataset can be of interest to environmental regulators and treatment plant operators.
• Building more potent statistical interference models for low sample tests (few exposure concentrations, limited amount of replication) within a high-throughput setup.
• It offers potential for integration into *in silico* tools (such as ToxCast pipeline, IVIVE).
• Further bioanalytical data supporting the EU Water Framework Directive reiteration and the incentive to include high-throughput bioassays into regulation.

## 2 Background

*In vitro* bioassays for the high-throughput assessment of environmental samples are gaining increasing attention from regulators and technical operators alike, given their potential to assess cumulative biological interference and to significantly support classical environmental analytical chemistry [1]. Currently, a key incentive for the scientific community is to integrate cellular bioanalytical tools into existing regulations, as proposed in the EU Water Framework Directive reiteration [2].

This dataset was primarily generated to assess the toxicological burden of organic micropollutants in selected WWTP-related samples using bioanalytical methods. As such, it can be regarded as a standard procedure in the emerging field. Second, the data presented here were utilised in an associated original research paper on a higher-tier level of evaluation, including semi-targeted, in silico-driven, effect-directed analysis and molecular docking modelling. For further details on the project’s concept and cross-referencing between the main article and *Data in Brief* co-submission, consult the supplementary manuscript (SM), Section 1. The higher-tier analyses in the original research article aim to improve bioanalytical tools further and address current shortcomings. Here, we aim to present the base-level bioanalytical data in a classical format. Thus, the base-level data is not concealed from readers of the associated article, but it may also appeal to an audience solely interested in the standard proceedings and results.

## 3 Data Description

For a simplified understanding of the following data description and material & methods sections, Table 1 provides a summary of the used abbreviations.

**Table 1:** Summary of used abbreviations.

Abbreviation	Explanation
A/B/C_X	Coding of environmental samples, see Table 4 for details
AhR	Aryl hydrocarbon receptor
ARNT	Aryl hydrocarbon receptor nuclear translocator protein
ARE	Antioxidant response element
Ctrl	Solvent control
DLR	Dual luciferase assay
DMSO	Dimethyl sulfoxide
FET	Fish embryo acute toxicity test
hpf	Hours post fertilisation
Keap1	Kelch-like ECH-associated protein 1
LOEC	Lowest observed effect concentration
LR	(single) luciferase assay
mFET	Modified zebrafish embryo test
MoA	Mode of action
MTS	Dimethyl-thiazol-sulfopheny tetrazolium-based cytotoxicity assay
NC	Negative control (solvent control)
NOEC	No observed effect concentration
Nrf2	Nuclear factor erythroid 2-related factor 2
REF	Relative enrichment factor
tBHQ	*tert*butylhydroquinone
TCDD	2,3,7,8-tetrachlorodibenzo-p-dioxin
SIx	Supplementary information, file no x
SM	Supplementary manuscript
WWTP	Wastewater treatment plant
XRE	Xenobiotic response element

Environmental water samples were collected from three WWTP sites located in southern and central Sweden (influent and effluent samples, see procedures in Section 4.1) and their respective recipients (upstream and downstream surface water). Subsequently, a multi-endpoint and multi-species bioanalytical assessment was performed. The entire dataset is presented in the following data description sections.

**Fig. 1:**
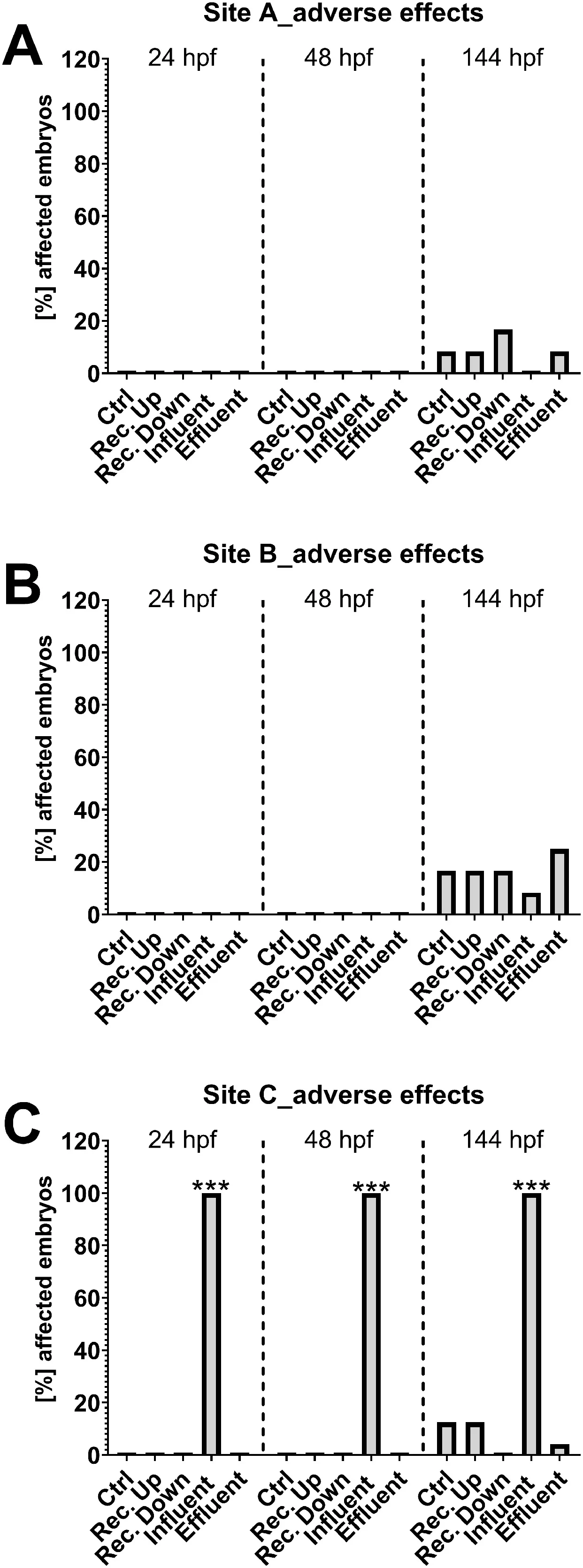
Lethal and sublethal (categorical) adverse effects were recorded via the mFET assay in a binary manner (affected vs. non-affected), summed up, and displayed above as a percentage of affected zebrafish embryos via bar plots. 12–24 embryos were exposed to the named environmental samples (vehicle control (Ctrl), recipient upstream (Rec. Up), recipient downstream (Rec. Down), influent, and effluent) derived from WWTPs at sites A, B, and C at a REF of 2. Endpoints were recorded at 24, 48, and 144 hpf. A one-sided, pairwise Fisher test with Benjamini-Hochberg adjustment was conducted to establish statistical significance versus control (***p < 0.001). Data and details are given in SI6.

**Fig. 2:**
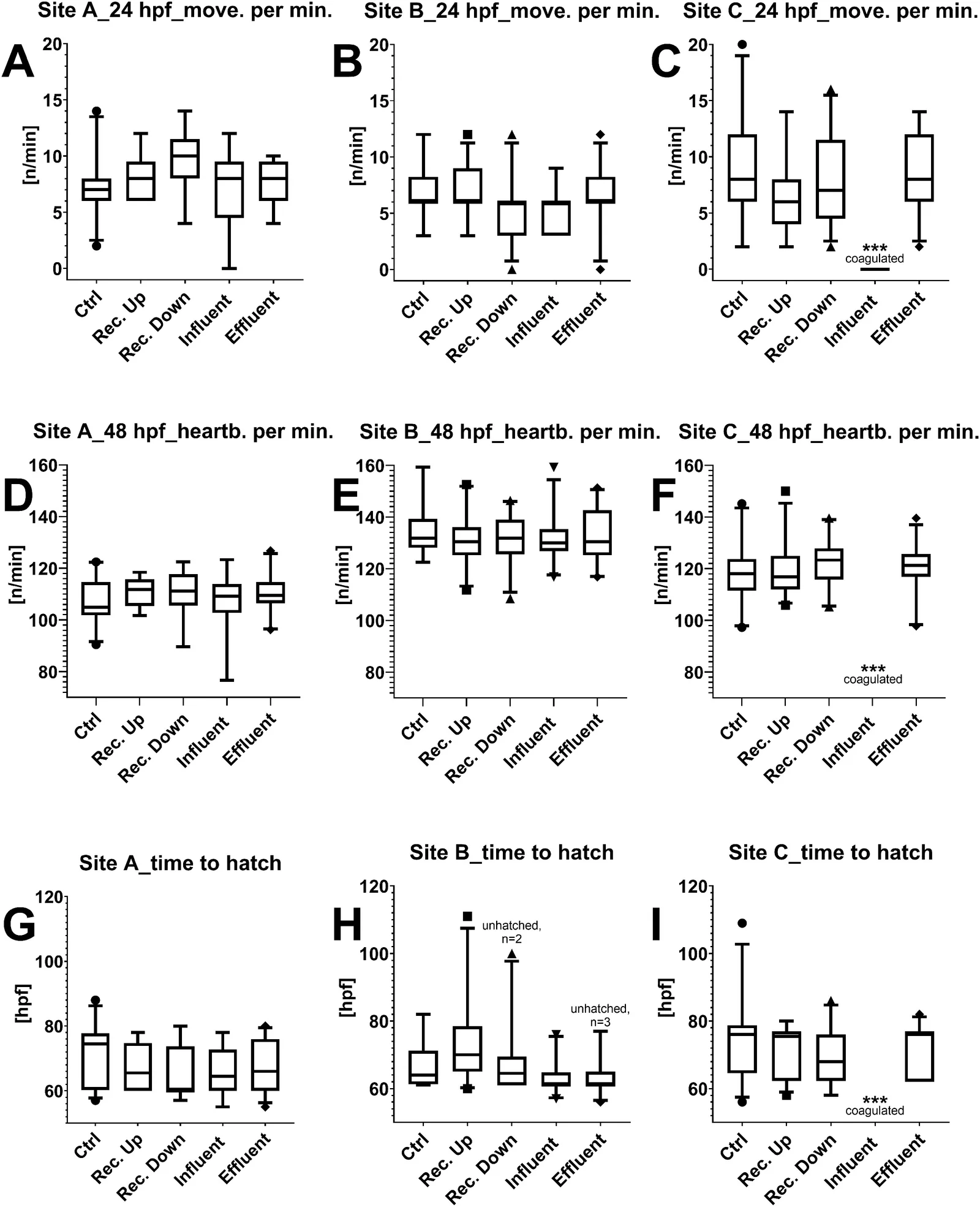
Continuous, sublethal endpoints recorded via the mFET assay in zebrafish embryos exposed to the named environmental samples from the investigated WWTPs sites A, B, and C at a REF of 2. 12–24 individuals were exposed to every named environmental sample (vehicle control (Ctrl), recipient upstream (Rec. Up), recipient downstream (Rec Down), influent, and effluent). Boxplots depict the numerical population of every sample and respective endpoint, recorded at 24 hpf (movements per minute, A-C), 48 hpf (heartbeats per minute, D-F), and the specific timepoint of hatching (G-I). Whiskers represent the 5th and 95th percentiles, respectively. Boxes indicate the 25th and 75th percentiles, with a line marking the median. Outliers are given as single dots. Statistical inference was investigated via two-sided Dunnett's tests (alpha level = 0.05). No statistically significant difference was observed between the means of the control and exposure groups. Numerical values compromised by lethality (coagulation) were given the value 0, and statistical inference versus control was computed by a two-sided, asymptotic Wilcoxon-Pratt signed-rank test (alpha level = 0.05; ***p < 0.001). Data and details are given in SI6.

All environmental samples were tested using a modified zebrafish embryo test (mFET; see Section 4.2 for methodological procedures and references). The dataset is depicted in an illustrative manner in Section 3.1. All raw data and underlying calculations are given in SI6 (data repository). First, a classical categorical assessment of apical lethal and sublethal endpoints (“adverse effects”) was conducted (Fig. 1). Second, additional non-lethal, numerical endpoints were investigated: movements per minute, heartbeats per minute, and time of hatch (Fig. 2). The respective assessment time in hours-post-fertilisation (hpf) is given in the individual graphs. Derived LOECs are summarised in Table 3.

**Fig. 3:**
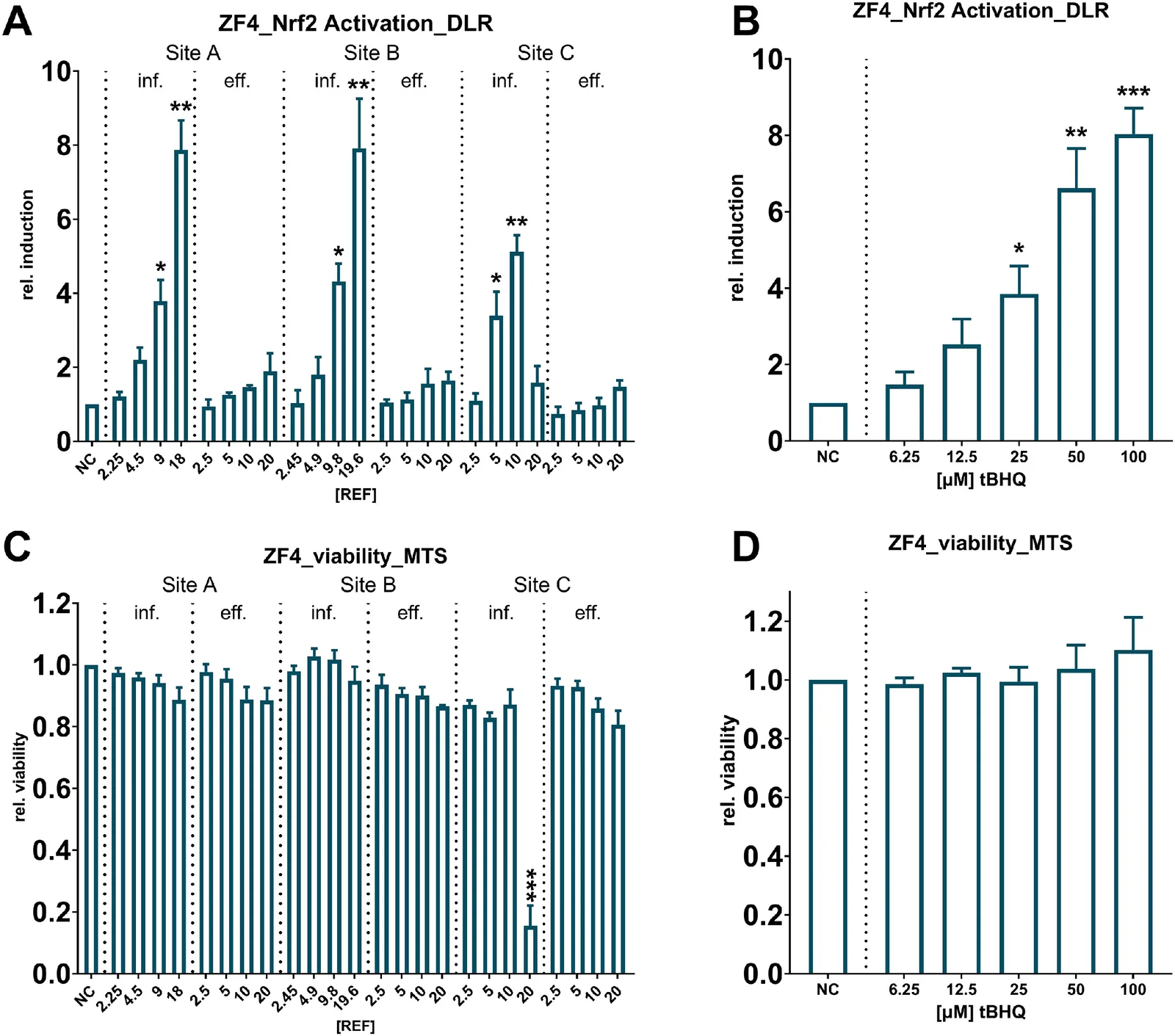
(A) Effects on luminescence measured in the zebrafish cell line ZF4 exposed to increasing concentrations of enriched environmental samples collected from WWTPs sites A, B, and C (influent (inf.); effluent (eff.)). Luminescence corresponds to quantitative Nrf2 transcription factor activation, measured via DLR assay in transiently transfected cells (see method section for details). Mean fold-normalised luminescence induction is illustrated as bars, with whiskers representing the SEM (experimental units n = 3; observational units N = 9). (B) Effects on luminescence measured in the zebrafish cell line ZF4 exposed to increasing concentrations of the reference compound *tert*butylhydroquinone (tBHQ). Luminescence corresponds to quantitative Nrf2 transcription factor activation, measured via DLR assay in transiently transfected cells (see method section for details). Mean fold-normalised luminescence induction is illustrated as bars, with whiskers representing the SEM (experimental units n = 3; observational units N = 9). (C, D) Cellular viability corresponds to control-normalised mitochondrial metabolism measured via the MTS assay. Each bar represents the mean, including SEM (experimental units n = 3; observational units N = 9). All statistical comparisons versus the negative vehicle control (NC) were conducted via one-sided Dunnett's tests (alpha level = 0.05; *p < 0.05, **p < 0.01, ***p < 0.001) on pooled raw data (see SI1 and SI7 for raw data and details on calculations).

**Fig. 4:**
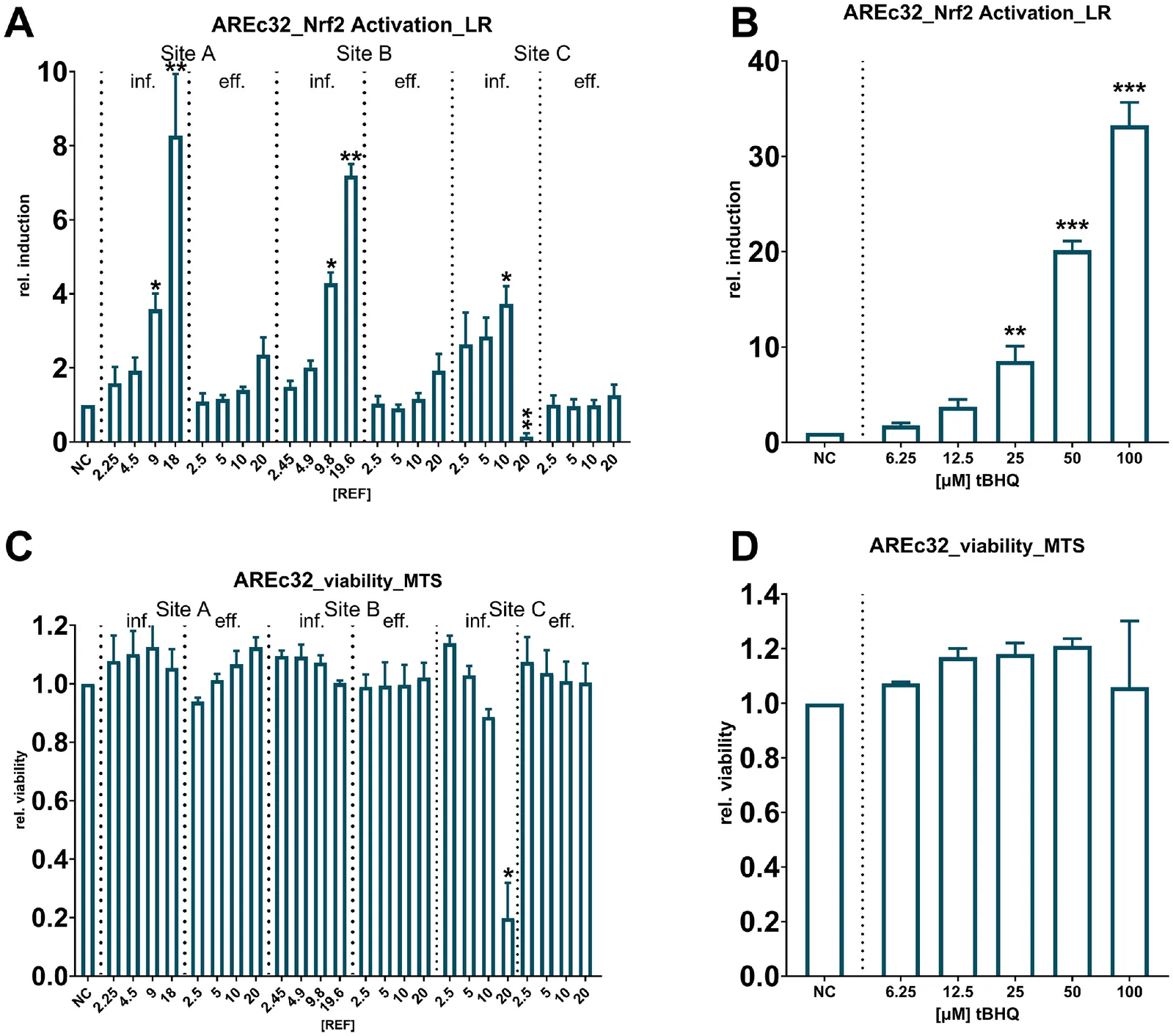
(A) Effects on luminescence measured in the human cell line MCF7/AREc32 exposed to increasing concentrations of enriched environmental samples collected from WWTPs sites A, B, and C (influent (inf.); effluent (eff.)). Luminescence corresponds to quantitative Nrf2 transcription factor activation, measured via LR assay in stably transfected cells (see method section for details). Mean fold-normalised luminescence induction is illustrated as bars, with whiskers representing the SEM (experimental units n = 3; observational units N = 10). (B) Effects on luminescence measured in the human cell line MCF7/AREc32 exposed to increasing concentrations of the reference compound *tert*butylhydroquinone (tBHQ). Luminescence corresponds to quantitative Nrf2 transcription factor activation, measured via LR assay in stably transfected cells (see method section for details). Mean fold-normalised luminescence induction is illustrated as bars, with whiskers representing the SEM (experimental units n = 4; observational units N = 12). (C, D) Cellular viability corresponds to control-normalised mitochondrial metabolism measured via the MTS assay. Each bar represents the mean, including SEM (experimental units n = 3; observational units N = 12). All statistical comparisons versus the negative vehicle control (NC) were conducted via one-sided Dunnett's tests (alpha level = 0.05; *p < 0.05, **p < 0.01, ***p < 0.001) on pooled raw data (see SI2 and SI7 for raw data and details on calculations).

**Fig. 5:**
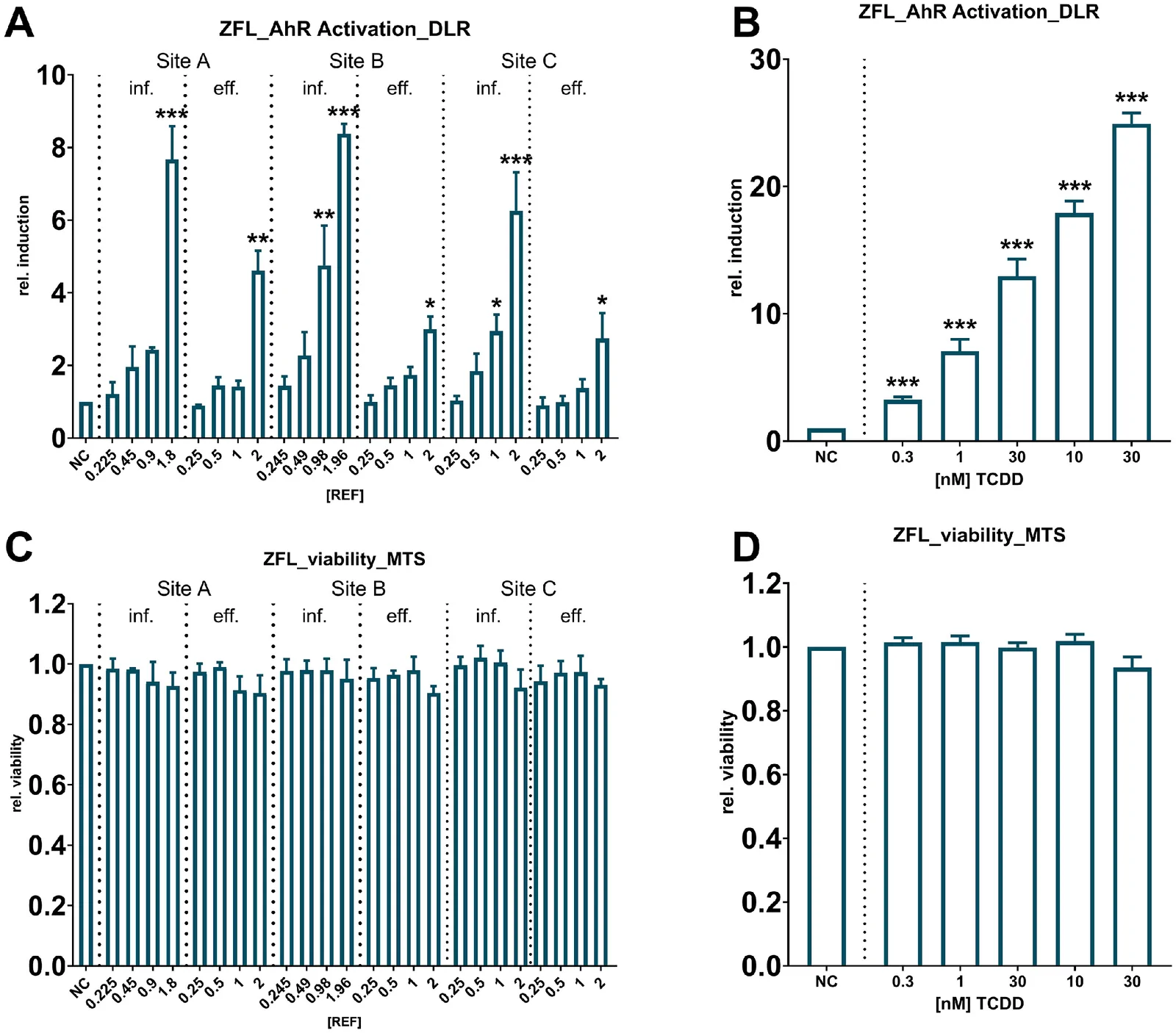
(A) Effects on luminescence measured in the zebrafish cell line ZFL exposed to increasing concentrations of enriched environmental samples collected from WWTPs sites A, B, and C (influent (inf.); effluent (eff.)). Luminescence corresponds to quantitative AhR transcription factor activation, measured via DLR assay in transiently transfected cells (see method section for details). Mean fold-normalised luminescence induction is illustrated as bars, with whiskers representing the SEM (experimental units n = 3; observational units N = 9). (B) Effects on luminescence measured in the zebrafish cell line ZFL exposed to increasing concentrations of the reference compound 2,3,7,8-tetrachlorodibenzo-p-dioxin (TCDD). Luminescence corresponds to quantitative AhR transcription factor activation, measured via DLR assay in transiently transfected cells (see method section for details). Mean fold-normalised luminescence induction is illustrated as bars, with whiskers representing the SEM (experimental units n = 4; observational units N = 12). (C, D) Cellular viability corresponds to control-normalised mitochondrial metabolism measured via the MTS assay. Each bar represents the mean, including SEM (experimental units n = 3; observational units N = 9). All statistical comparisons versus the negative vehicle control (NC) were conducted via one-sided Dunnett's tests (alpha level = 0.05; *p < 0.05, **p < 0.01, ***p < 0.001) on pooled raw data (see SI3 and SI7 for raw data and details on calculations).

**Fig. 6:**
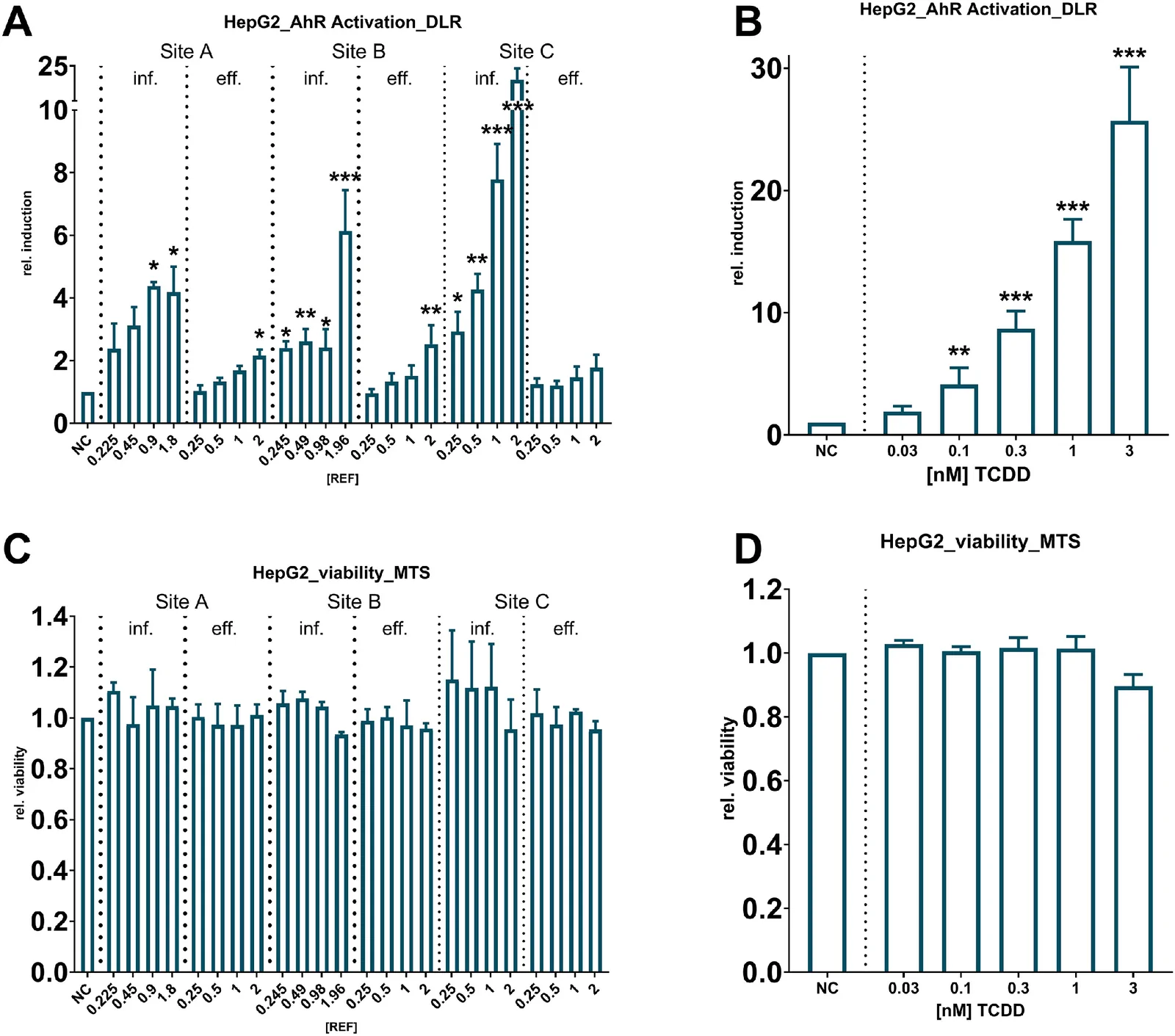
(A) Effects on luminescence measured in the human cell line HepG2 exposed to increasing concentrations of enriched environmental samples collected from WWTPs sites A, B, and C (influent (inf.); effluent (eff.)). Luminescence corresponds to quantitative AhR transcription factor activation, measured via DLR assay in transiently transfected cells (see method section for details). Mean fold-normalised luminescence induction is illustrated as bars, with whiskers representing the SEM (experimental units n = 3; observational units N = 9). (B) Effects on luminescence measured in the human cell line HepG2 exposed to increasing concentrations of the reference compound 2,3,7,8-tetrachlorodibenzo-p-dioxin (TCDD). Luminescence corresponds to quantitative AhR transcription factor activation, measured via DLR assay in transiently transfected cells (see method section for details). Mean fold-normalised luminescence induction is illustrated as bars, with whiskers representing the SEM (experimental units n = 4; observational units N = 12). (C, D) Cellular viability corresponds to control-normalised mitochondrial metabolism measured via the MTS assay. Each bar represents the mean, including SEM (experimental units n = 3; observational units N = 9). All statistical comparisons versus the negative vehicle control (NC) were conducted via one-sided Dunnett's tests (alpha level = 0.05; *p < 0.05, **p < 0.01, ***p < 0.001) on pooled raw data (see SI3 and SI7 for raw data and details on calculations).

**Fig. 7:**
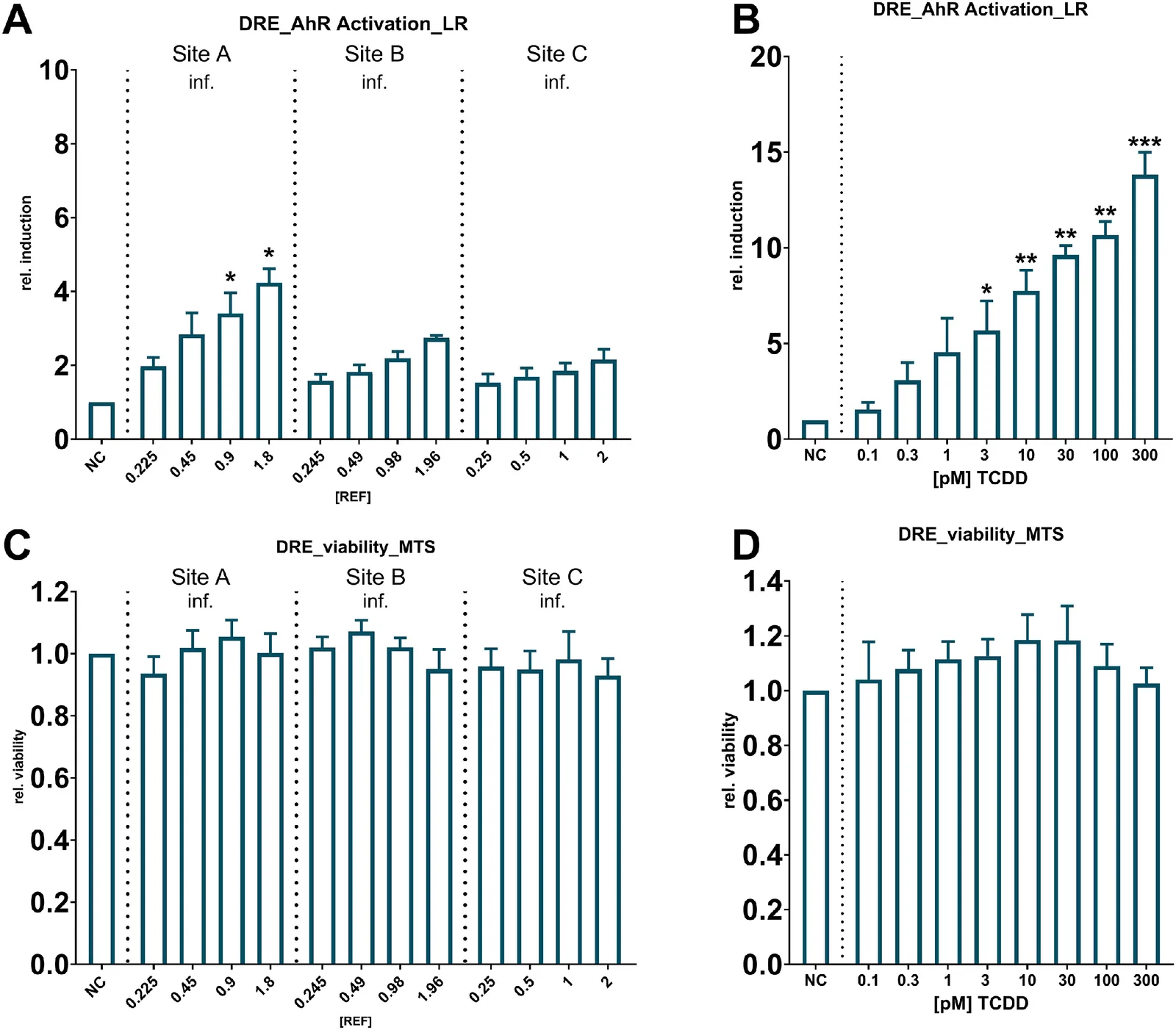
(A) Effects on luminescence measured in the mouse cell line Hepa1c1c7/DREcoScreen exposed to increasing concentrations of enriched environmental samples collected from WWTPs sites A, B, and C (influent (inf.)). Luminescence corresponds to quantitative AhR transcription factor activation, measured via LR assay in stably transfected cells (see method section for details). Mean fold-normalised luminescence induction is illustrated as bars, with whiskers representing the SEM (experimental units n = 5; observational units N = 17). (B) Effects on luminescence measured in the mouse cell line Hepa1c1c7/DREcoScreen exposed to increasing concentrations of the reference compound 2,3,7,8-tetrachlorodibenzo-p-dioxin (TCDD). Luminescence corresponds to quantitative AhR transcription factor activation, measured via LR assay in stably transfected cells (see method section for details). Mean fold-normalised luminescence induction is illustrated as bars, with whiskers representing the SEM (experimental units n = 5; observational units N = 20). (C, D) Cellular viability corresponds to control-normalised mitochondrial metabolism measured via the MTS assay. Each bar represents the mean, including SEM (experimental units n = 3–5; observational units N = 10–17). All statistical comparisons versus the negative vehicle control (NC) were conducted via one-sided Dunnett's tests (alpha level = 0.05; *p < 0.05, **p < 0.01, ***p < 0.001) on pooled raw data (see SI5 and SI7 for raw data and details on calculations).

Further, influent and effluent samples were analysed with cellular reporter gene assays of three different species (zebrafish, human, and mouse, see Section 4.3 for methodological procedures and references), covering the oxidative stress (Fig. 3, Fig. 4) and xenobiotic metabolism (Figs. 5, 6, and 7) modes of action (MoAs). The dataset is depicted in an illustrative manner in Section 3.2. All raw data and underlying calculations are given in SI1–5 and SI7. The derived LOECs are summarised in Table 3. In addition to the luciferase reporter-driven MoA output, cellular viability was tested from the identical technical replicates by multiplexing with the MTS assay (see Section 4.4.3 and 4.5) to ensure that recorded effects are not impacted by cytotoxicity (Figs. 3–7, panels C). A positive inducer reference compound was tested in parallel to the environmental samples on every plate. The summarised data are given in Figs. 3–7, panels B, including the viability assessment in panels D. A cytotoxic effect was only encountered once for sample C_I at REF 20.

**Fig. 8:**
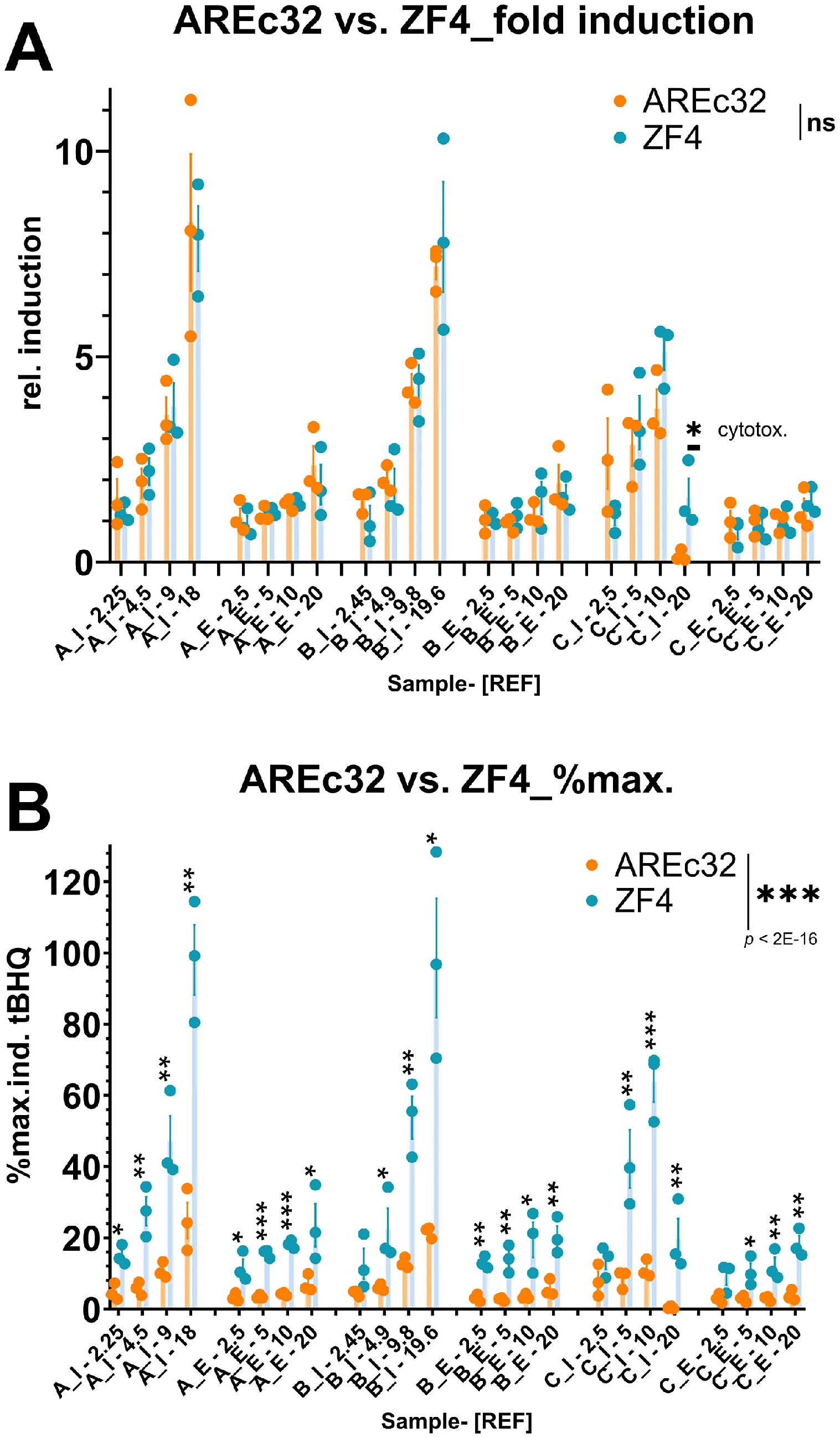
Overall and pairwise comparison of the measured luminescence of the oxidative stress MoA in ZF4 and AREc32 cells (data from Fig. 3, Fig. 4) from WWTPs sites A, B, and C (influent (I), effluent (E)). Data are depicted as either fold-normalised or maximum induction adjustment to the reference compound *tert*butylhydroquinone (tBHQ). Bars represent the overall means, with the whiskers indicating the SEM. Dots represent the means of single experiments (experimental units n = 3). The respective colour coding is depicted in the graph. A two-way ANOVA with exposure concentration and cell line as factors was conducted. The cell line variance results are given in the upper right corner, including the computed p-value. Effect means' variances between cell lines were calculated via two-sided Welch's two-sample t-tests with Benjamini-Hochberg adjustment for family-wise error rate (alpha level = 0.05; *p < 0.05, **p < 0.01, ***p < 0.001, ns=non-significant) for respective comparisons. For details on data handling and calculations, see also SI8.

**Fig. 9:**
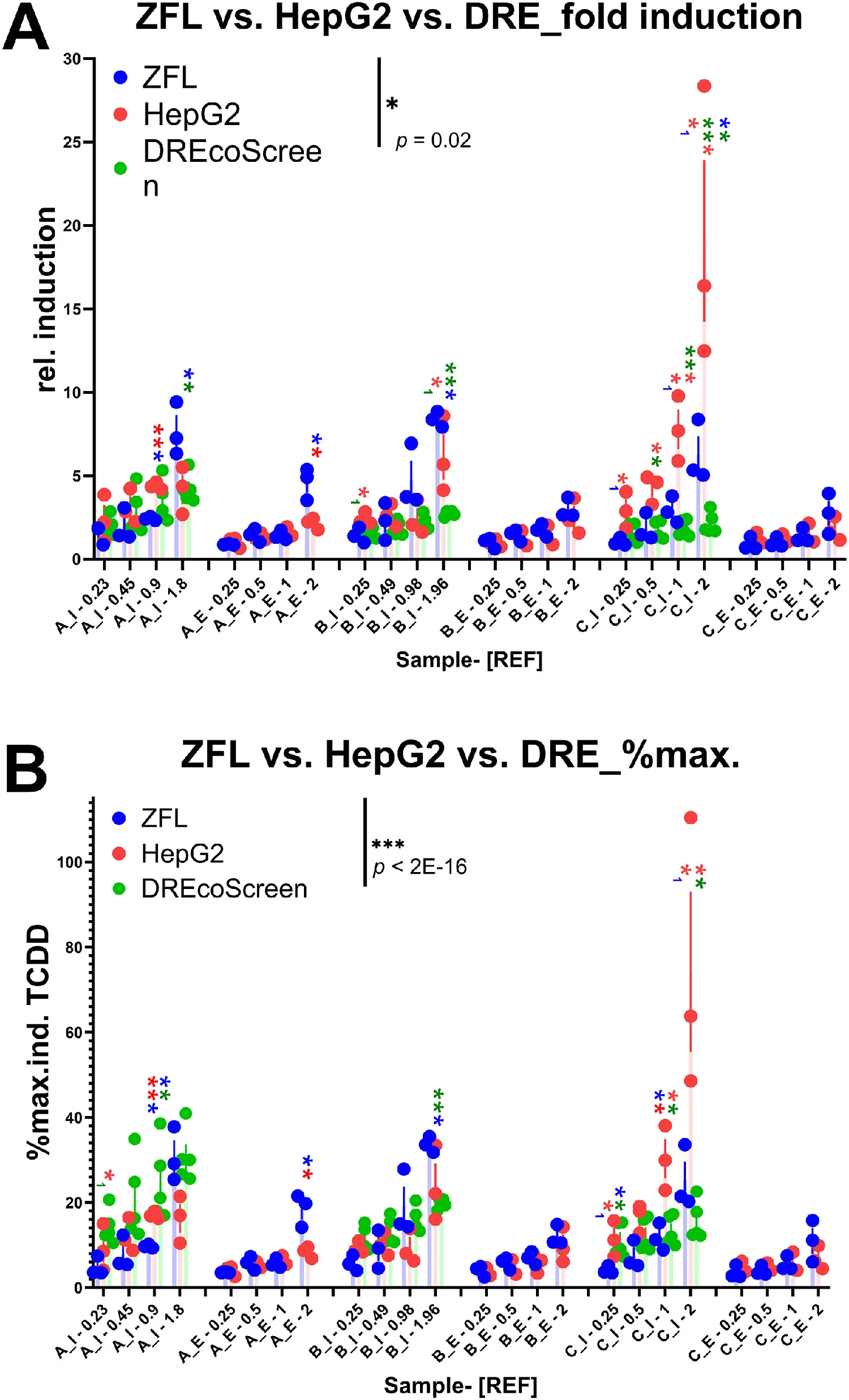
Overall and pairwise comparison of the measured luminescence of the xenobiotic metabolism MoA in ZFL, HepG2, and DREcoScreen cells (data from Figs. 5, 6, and 7) from WWTPs sites A, B, and C (influent (I), effluent (E)). Data are depicted as either fold-normalised or maximum induction adjustment to the reference compound 2,3,7,8-tetrachlorodibenzo-p-dioxin (TCDD). Bars represent the overall means, with the whiskers indicating the SEM. Dots represent the means of single experiments (experimental units n = 3–5). The respective colour coding is depicted in the graph. A two-way ANOVA with exposure concentration and cell line as factors was conducted. The cell line variance results are given in the upper left corner, including the computed p-value. Effect means' variances between cell lines were calculated via two-sided Welch's two-sample *t*-tests with Benjamini-Hochberg adjustment for family-wise error rate (alpha level = 0.05; *p < 0.05, **p < 0.01, ***p < 0.001) for respective comparisons. The coloured asterisks indicate pairwise comparisons between two specified cell lines. An additionally coloured number denotes the other cell line in a one-asterisk scenario. For details on data handling and calculations, see also SI8.

Additionally, a comparison between cell lines and species was conducted for the investigated MoAs. The data were expressed both as fold-induction (Fig. 8, Fig. 9, panels A) and as a percentage of the maximum reference induction (Fig. 8, Fig. 9, panels B). Analysed data and calculations are accessible in SI8.

The raw and analysed data, including data transformations and calculations, are given in the supplementary information (SI) and uploaded to the data-sharing repository (https://doi.org/10.5878/zwnq-qb88). The SIs are numbered SI1 to SI8; Table 2 also summarises the SIs. The illustrations below reference each raw and analysed dataset to guide the reader and facilitate navigation.

**Table 2:** Description of supplementary information (SI) in the data repository.

Name	Content	Associated illustrations
SI1_ZF4_Nrf2	Raw and analysed data of all experiments conducted with the ZF4 assay	Fig. 3, Fig. 8
SI2_MCF_Nrf2	Raw and analysed data of all experiments conducted with the MCF7/AREc32 assay	Fig. 4, Fig. 8
SI3_ZFL_AhR	Raw and analysed data of all experiments conducted with the ZFL assay	Fig. 5, Fig. 9
SI4_HepG2_AhR	Raw and analysed data of all experiments conducted with the HepG2 assay	Fig. 6, Fig. 9
SI5_DREco_AhR	Raw and analysed data of all experiments conducted with the DREcoScreen assay	Fig. 7, Fig. 9
SI6_mFET	Raw, analysed data, and statistical interference of the mFET assay	Fig. 1, Fig. 2
SI7_Rcalc	Transformation and statistical interference conducted in all cellular bioassays	Figs. 3–7
SI8_Rcalc_vs	Transformation and statistical interference conducted for all pairwise comparisons between assays/species	Fig. 8, Fig. 9
SM	Supplementary manuscript	-

### 3.1 Bioanalytical assessment of environmental samples via the mFET assay (raw data and analysis in SI6)

### 3.2 Bioanalytical assessment of environmental samples via multi-species cellular reporter-gene assays (raw data and analysis in SI1–5 and SI7)

### 3.3 LOEC summary of the entire bioanalytical assessment

**Table 3:** Lowest observed effect concentrations (LOEC; not applicable (n.a.) - no LOEC computed; not tested (n.t.)) in all assays from WWTPs sites A, B, and C (influent (I), effluent (E)), values depict concentrations of environmental samples as relative enrichment factor [REF].

Site	mFET	ZF4	AREc32	ZFL	HepG2	DRE
A_I	n.a.	9	9	1.8	0.9	0.9
A_E	n.a.	n.a.	n.a.	2	2	n.t.
B_I	n.a.	9.8	9.8	0.98	0.245	n.a.
B_E	n.a.	n.a.	n.a.	2	2	n.t.
C_I	2	5	10	1	0.25	n.a.
C_E	n.a.	n.a.	n.a.	2	n.a.	n.t.

### 3.4 Inter-species/assay comparison of the investigated MoAs

## 4 Experimental Design, Materials and Methods

The experimental design, materials, and methods section is divided into three parts: environmental sample preparation and extraction, effect-based sample assessment via the mFET, and effect-based sample assessment via various reporter-gene cell lines. Methodologies concerning the entire project are only briefly mentioned herein, with detailed descriptions provided in the SM, Section 2.

### 4.1 Environmental sample preparation and extraction

Water samples were collected from three WWTPs (sites A to C) in Sweden that are of anthropogenic and ecological concern (fig. S1 in SM). Every site included influent and effluent WWTP samples, as well as surface water samples taken upstream and downstream of the associated recipient stream. The SM, Section 2.1, provides more details regarding the environmental samples and WWTP characteristics (tab. S3).

Environmental sample extraction was previously described in detail by Golovko et al. [3]. Technical details can be found in either the above-named article or in the SM, Sections 2.1 and 2.2. Enrichment and dilution factors were calculated as described by Escher et al. [4].

### 4.2 Modified fish embryo test (mFET)

#### 4.2.1 Zebrafish husbandry and egg collection

Adult stock zebrafish (*Danio rerio*) originated from a wild-type strain purchased from a local supplier (Akvarie Hobby, Uppsala, Sweden). Fish were maintained in the aquatic facility for several generations in a carbon-filtered tap-water flow-through system at 26 ± 1 °C, a 12/12 LD photoperiod, pH 7.9, hardness 6.7 °dH, and a conductivity of 468 µS/cm. The following feed was applied diurnally: flakes (Sera Vipan, Heinsberg, Germany), freeze-dried chironomids (Nutrafin-Hagen, Holm, Germany), and frozen artemia nauplii (Akvarie Teknik, Filipstad, Sweden). To induce spawning, 10–14 adult zebrafish were placed in stainless steel mesh cages within four 10 L aquaria the previous afternoon. At the onset of light, zebrafish were allowed to spawn for 30 min. Eggs were collected and pooled from all aquaria, rinsed of debris, and transferred to Petri dishes filled with carbon-filtered tap water (see above).

#### 4.2.2 Bioanalytical assessment via the modified fish embryo test (mFET)

The mFET is an alternating version of the fish embryo acute toxicity test (FET, OECD TG 236 [5]), considering time points beyond 96 hpf up to 144 hpf. The mFET is not considered animal testing according to Swedish legislation. The mFET procedure has been described in detail by Carlsson et al. [6]. In short, the test adds to the standard FET apical acute toxicity endpoints, namely coagulation, lack of heartbeat, lack of somite formation, and non-detachment of tails, several sublethal categorical and continuous endpoints, which are recorded differently at 24 and 48 hpf (consult Table 4 for details).

**Table 4:** Observed endpoints within the modified fish embryo test (mFET) at 24, 48, and 144 hpf. Modified from Carlsson et al. (2013).

Endpoints	Description	24 hpf	48 hpf	144 hpf
***Lethal categorical***				
Coagulation	The embryo is coagulated with no structures	X	X	X
Lack of heartbeats	The embryo has no visible heartbeat		X	X
***Sublethal categorical***				
Tail deformation	The tail is shorter than usual or curved and non-detached	X	X	X
Lack of somite	Lack of somite formation	X	X	X
Eye deformation	The eye is not ordinarily round with a visible lens	X	X	X
Head deformation	The head is not normally developed	X	X	X
Reduced pigmentation	The embryo shows reduced pigmentation		X	
Oedema	Oedema is present		X	X
Tremor	The embryo shows tremor		X	X
Side-lying	In relaxed mode, the embryo lies on its side			X
Unhatched	The embryo is alive but has not hatched at the observation time			X
***Sublethal continuous***				
Movements	The number of movements is counted for 30 s and extrapolated to movements per minute	X		
Heart rate	Time for 30 heartbeats is recorded and converted to beats per minute		X	
Time to hatching	Time of the first 1 h-interval photo when the embryo hatches		48–144 hpf	

For this experiment, carbon-filtered tap water was aerated for 30 min before the environmental sample was added. A volume of 20 µL from each of the 2000x-concentrated environmental samples was added to 19.98 mL of carbon-filtered tap water, resulting in a final REF of 2 and a DMSO content of 0.1% (v/v). A vehicle control group (negative control) containing only 0.1% (v/v) DMSO was prepared in parallel.

After spawning, 30 eggs per group were transferred to Petri dishes containing the respective environmental samples (RU, RN, E, and I from sites A, B, and C, and respective controls). Within 15 min after the spawning event, collected zebrafish eggs were checked for fertilisation (approximately 4-cell stage) under the stereo microscope (Leica EZ4D, 8–35x magnification, Wetzlar, Germany) and individually transferred to a transparent 96-well plate (Corning, New York, USA), containing 250 µL/well of the identical environmental sample. From each environmental sample and control, 12–24 embryos were taken. Within the 96-well plates, the exposure setup was arranged in a semi-randomised (diagonal) block design, minimising possible edge effects which might affect measured endpoints (see SI6 for plate setups). During static exposure up to 144 hpf, 96-well plates were kept at 26 ± 1 °C and covered with parafilm to eliminate contamination and evaporation.

According to the mFET assay (Table 4), lethal and sublethal endpoints were recorded under the stereo microscope at 24, 48, and 144 hpf. Categorical endpoints were scored in a binary manner (encountered/not encountered). Numerical, continuous endpoints were derived by extrapolating manual counts. The hatching time was recorded using a time-lapse camera (Canon EOS 500D), which automatically photographed the plates hourly between 48 and 144 hpf.

#### 4.2.3 Statistical analysis of mFET data and plotting

Residuals of numerical, continuous values were checked for at least semi-normality and homogeneity of variance. First, by visually inspecting the respective data sets' residuals via histogram, boxplots, fitted vs. actual residuals, normal Q-Q plot, and population density plot. Second, statistically, by Shapiro-Wilk and Levene's tests. Upon meeting parametric criteria, a two-sided Dunnett's test (alpha level = 0.05) was conducted to assess statistical significance relative to the control group. If the criteria were not met, a non-parametric Kruskal-Wallis test followed by a two-sided Dunn's post hoc test (alpha level = 0.05) with Benjamini-Hochberg adjustment was conducted for the respective dataset. To account for lethality in numerical outcomes, all such endpoints were assigned a value of 0. Specifically, affected subgroups were tested versus control via a two-sided, asymptotic Wilcoxon-Pratt signed-rank test (alpha level = 0.05), allowing ties and heteroscedastic variance.

All categorical results were summed in a binary manner (affected vs. non-affected) for each specific time point of investigation. Statistical significance relative to the control was assessed using a one-sided (lesser) pairwise Fisher's test with Benjamini-Hochberg adjustment.

Statistical inference and visual inspection were conducted in R Studio (version 2023.03 *Cherry Blossom*; Posit Team, Boston, USA), using the R core packages (version R.4.3.0; R Core Team, Vienna, Austria) and additional scripts provided by Green et al. [7]. All data and tests are available in SI6. The final plotting of endpoints was conducted in GraphPad Prism 9 (GraphPad Software, Boston, USA). Continuous endpoints were plotted as boxplots, whereas categorical endpoints were plotted as bar charts.

### 4.3 Bioanalytical assessment via reporter gene assays

Influent and effluent samples of the three investigated WWTPs were also analysed via five different reporter gene assays, covering two MoAs (activation of oxidative stress defence via Nrf2-Keap1-ARE interaction and activation of the xenobiotic metabolism via AhR-ARNT-XRE interaction) in cells from three different species (zebrafish, human, and mouse). Reporter gene cell line specifications and subsequent experimental setups are briefly described in the following sections and in detail in the SM, Section 2.3.

#### 4.3.1 Oxidative stress reporter assays

The zebrafish oxidative stress response assay is based on the embryonic fibroblast cell line ZF4 (CVCL_3275) [8], transiently transfected with an Nrf2-responsive reporter construct [9,10]. The human oxidative stress response assay MCF7/AREc32 reporter line (CVCL_1D32) [11] was obtained from Ximbio (London, UK). Details on the reporter constructs, applications, and culture conditions are given in the SM, Sections 2.3.1 and 2.3.2.

#### 4.3.2 Xenobiotic metabolism reporter assays

The zebrafish xenobiotic metabolism response reporter assay is based on the hepatocyte cell line ZFL (CVCL_3276) [12], transiently transfected with an AhR-responsive reporter construct, containing a zebrafish-optimised endogenous genomic reporter [13]. The human xenobiotic metabolism response reporter assay is based on the hepatoma cell line HepG2 (CVCL_0027) [14], transiently transfected with an AhR-responsive reporter construct (pGudLuc7.5) [15]. The mouse xenobiotic metabolism response reporter assay DREcoScreen (DRE) (CVCL_EG66) [16] was obtained from Hiro Biotech (Singapore) via the Japanese Collection of Research Bioresources (Ibaraki, Osaka, Japan). Details on the reporter constructs, applications, and culture conditions are given in the SM, Sections 2.3.3 to 2.3.5.

### 4.4 Reporter gene bioassays: cell handling, transgenesis, exposure, and readout

The experiments spanned a duration of three (stable reporter) to four (transient reporter) days, which multiplexed the MoA luciferase endpoint readout with cellular viability (cytotoxicity) assessment. Each experiment was replicated at least thrice. The following paragraphs outline the procedures for the reporter gene bioassay. For a detailed description, please consult the SM, Sections 2.3.6 to 2.3.11.

#### 4.4.1 Cell plating

Cells were seeded onto white, clear-bottom 96-well microtiter plates (Corning, New York, USA) at varying cell concentrations specific to each cell line (ZF4, 2.5 × 10^5^ n/mL; MCF7/AREc32, 1.5 × 10^5^ n/mL; ZFL, 1.5 × 10^5^ n/mL; HepG2, 1.5 × 10^5^ n/mL; DRE, 10^5^ n/mL), and returned to the incubator for 24 h. The recombinant transgenesis protocols for ZF4, ZFL, and HepG2-based reporter cell lines were performed as described in the SM, Sections 2.3.1, 2.3.3, 2.3.4, and 2.3.7 or in previous publications [9,10,13,17].

#### 4.4.2 Exposure

For the oxidative stress assays (ZF4 and MCF7/AREc32), cells were exposed to influent and effluent samples at REF 2.5, 5, 10, and 20. For the xenobiotic metabolism assays (ZFL, HepG2, and DRE), cells were exposed at REF 0.25, 0.5, 1, and 2. Notably, DRE cells were only exposed to influent samples. Experimental and technical replication units are given in the captions of the respective graphs. For more details on used controls and technical replication per experiment, consult the SM, Section 2.3.8.

In addition to the WWTP samples, reference compounds were applied to every plate. tert‑Butylhydroquinone (tBHQ; CAS 1948–33–0; Sigma-Aldrich, St. Louis, USA) was utilised as a reference compound of the oxidative stress MoA, whereas 2,3,7,8-tetrachlorodibenzo-p-dioxin (TCDD; CAS 1746–01–6; Supelco analytical standard; Sigma- Aldrich, Steinheim, Germany) was used for the xenobiotic metabolism. The following titration steps were applied: tBHQ, linear scale, 6.25 to 100 μM, for both ZF4 and MCF7/AREc32. TCDD was titrated in half-log steps across different potency ranges for the assays used, reflecting species-specific potencies: ZFL: 0.3 to 30 nM; HepG2: 0.03 to 3 nM; DRE: 0.1 to 300 pM.

#### 4.4.3 Multiplexed readouts

After 24 h exposure, both cytotoxicity and the reporter-related luciferase readouts were acquired from the same plate by multiplexing a tetrazolium-salt-based “MTS”-assay (CellTiter 96® AQueous One Solution Cell Proliferation Assay; Promega, Madison, USA) with the luciferase (stable reporter) or Dual-Luciferase® (transient reporter; Promega, Madison, USA) reporter assay. MTS-turnover was measured at 490 nm absorbance, and luminescence units were measured following a flash luminescence protocol, both on a Tecan Spark plate reader (Tecan, Männedorf, Switzerland). For more details on readout procedures, consult the SM, Sections 2.3.9 to 2.3.11.

### 4.5 Reporter gene bioassays: statistical analysis and plotting

DLR-related raw luminescence data were first normalised to cell number and transfection efficiency by dividing the primary Firefly luciferase (Fluc) signal by the constitutive Renilla luciferase (Rluc) signal for every technical replicate. Single-reporter assay (LR) raw luminescence data were taken as measured. Raw absorbance units recorded via the MTS assay were handled similarly to the single-luciferase outputs. Technical replicates from every exposure group were pooled to obtain experimental means. Statistical analysis was conducted on pooled means of three to five experiments per setup (final experimental units n = 3 – 5; final observational units N = 9 – 20). For visualisation and comparison, luciferase activity and viability were also expressed as fold-change relative to the controls (see SI1–5 for raw data and details).

For each assay, pooled experimental mean data were tested for normality and variance homogeneity of residuals to meet parametric criteria (see SI7). Normality, or at least semi-normality, of the residuals was assessed using the Shapiro-Wilk test, and homogeneity of variance using Levene's test. Visual inspection was conducted using histograms, box plots, fitted vs actual residuals, normal Q-Q plots, and population density plots. If the criteria were not met, the data were either log(10)- or Box-Cox-transformed and re-assessed (see SI7 for details). Upon meeting parametric criteria, a one-sided Dunnett's test (alpha level = 0.05) was conducted to assess statistical significance relative to the control group. All statistical tests and visual inspections were performed in R Studio (version 2023.03 *Cherry Blossom*; Posit Team, Boston, USA), using the R core packages (version R.4.3.0; R Core Team, Vienna, Austria) and additional scripts provided by Green et al. [7]. Plotting was conducted in GraphPad Prism 9 (GraphPad Software, Boston, USA) using fold-change-normalised data. All results were plotted as bar charts.

To compare the effects of various environmental samples across cell lines, fold-normalised luminescence data for the respective REF exposure concentrations were subjected to the same data-handling procedures as mentioned above to meet parametric test criteria (see also SI8). Beyond, the data were normalised to the maximum response of the respective reference compound (tBHQ or TCDD) to account for differences in species sensitivity. Subsequently, the data were treated identically to the fold-induction data and handled in parallel. An overall effect on the used cell line was tested via a two-way ANOVA with both exposure concentration and used reporter line as factors. Two-way ANOVAs were conducted for the oxidative stress (AREc32 vs. ZF4) and the xenobiotic metabolism (DRE vs. ZFL vs. HepG2) MoAs. Additionally, in-between-comparison tests were performed to assess specific effects across cell lines for each exposure concentration. Therefore, two-sided Welch's two-sample t-tests (alpha level = 0.05) with Benjamini-Hochberg adjustment for family-wise error rate were employed to compare cell lines (details in SI8). All calculations were conducted in R Studio (version 2023.03 *Cherry Blossom*; Posit Team, Boston, USA), using R core packages (version R.4.3.0; R Core Team, Vienna, Austria) and an additional script provided by Green et al. [7]. Plotting was performed in GraphPad Prism 9, and all results were plotted as bar charts.

## Limitations

None.

## Ethics Statement

Not applicable; the manuscript does not contain clinical studies, patient data, or animal experiments. All authors acknowledged and followed the ethical requirements for publication in Data in Brief.

## CRediT Author Statement

**SLM**: conceptualisation, methodology, software, validation, formal analysis, investigation, data curation, writing - original draft, writing - review & editing, visualisation, project administration, funding acquisition; **GM**: validation, formal analysis, investigation, data curation, writing - review & editing; **OG**: methodology, validation, investigation, writing - review & editing; **KF**: validation, formal analysis, investigation, data curation, writing - review & editing; **SA**: validation, investigation; **SÖ**: methodology, resources, writing - review & editing, supervision, project administration, funding acquisition; **LA**: methodology, resources, writing - review & editing, supervision, project administration, funding acquisition; **JL**: conceptualisation, methodology, resources, writing - review & editing, supervision, project administration, funding acquisition

## Data Availability

Researchdata.se - Swedish National Data ServiceDataset connected to the project “Cross-species bioanalytical assessment of the oxidative stress and xenobiotic metabolism modes of action for environmental water samples” (Original data).

## Appendix Supplementary materials

Image, application 1
